# A Novel General Imaging Formation Algorithm for GNSS-Based Bistatic SAR

**DOI:** 10.3390/s16030294

**Published:** 2016-02-26

**Authors:** Hong-Cheng Zeng, Peng-Bo Wang, Jie Chen, Wei Liu, LinLin Ge, Wei Yang

**Affiliations:** 1 School of Electronic and Information Engineering, Beihang University, Beijing 100191, China; zenghongcheng@buaa.edu.cn (H.-C.Z.); wangpb7966@163.com (P.-B.W.); yangweigigi@sina.com (W.Y.); 2 Collaborative Innovation Center of Geospatial Technology, Wuhan 430079, China; 3 Electronic and Electronic Engineering Department, University of Sheffield, Sheffield S1-3JD, UK; w.liu@sheffield.ac.uk; 4 School of Civil & Environment Engineering, The University of New South Wales (UNSW), Sydney NSW 20512, Australia; l.ge@unsw.edu.au

**Keywords:** bistatic SAR, GNSS, bulk RCMC, modified hybrid correlation

## Abstract

Global Navigation Satellite System (GNSS)-based bistatic Synthetic Aperture Radar (SAR) recently plays a more and more significant role in remote sensing applications for its low-cost and real-time global coverage capability. In this paper, a general imaging formation algorithm was proposed for accurately and efficiently focusing GNSS-based bistatic SAR data, which avoids the interpolation processing in traditional back projection algorithms (BPAs). A two-dimensional point target spectrum model was firstly presented, and the bulk range cell migration correction (RCMC) was consequently derived for reducing range cell migration (RCM) and coarse focusing. As the bulk RCMC seriously changes the range history of the radar signal, a modified and much more efficient hybrid correlation operation was introduced for compensating residual phase errors. Simulation results were presented based on a general geometric topology with non-parallel trajectories and unequal velocities for both transmitter and receiver platforms, showing a satisfactory performance by the proposed method.

## 1. Introduction

Bistatic Synthetic Aperture Radar (SAR) plays an important role in various remote sensing applications, where the transmitter could be a dedicated SAR [1,2,3] or opportunistic illuminator systems, such as communication satellites or global navigation satellite systems (GNSS). Due to its safety, convenience and low cost, bistatic SAR using transmitters of opportunity has attracted more and more attention and developed very quickly, both theoretically and practically, over the past few years [4,5,6,7,8].

Our research focuses on the GNSS-based bistatic SAR, as the GNSS system with a designed permanent global coverage is an ideal choice for bistatic SAR compared with other opportunistic illuminators. Reflected GNSS signals can be utilized to image the interesting area as an innovative, all-time and all-weather microwave (L-band) remote sensing tool [9,10]. In addition, GNSS constellations consist of over 100 satellites (GPS, GLONASS, Galileo and BeiDou), and multi-angle observation from over 20 satellites in any given area is attainable [11], which could potentially be used for increasing the imaging formation space [12,13,14,15]. Furthermore, GNSS-based bistatic SAR can achieve easy synchronization aided by the GNSS time service [16,17].

For imaging, many modified algorithms for bistatic SAR with specific geometric configurations have been presented [18,19,20,21,22], such as the range Doppler algorithm (RDA), the wavenumber domain algorithm (WDA), the back-projection algorithm (BPA), the chirp scaling algorithm (CSA), *etc.* For GNSS-based bistatic SAR, however, none of the above-mentioned algorithms is applicable due to its unique topology and non-designed radar signal. Based on the assumption of parallel flight paths of transmitter and receiver, a modified RDA is proposed in [23], and a modified BPA is presented in [16], which has a high computational complexity for interpolation. Due to the unique range history, no frequency domain algorithm without any approximation has been proposed yet in the literature. On the other hand, the hybrid correlation algorithm is an accurate and flexible algorithm by performing two-dimensional (2-D) correlation between the echo signal and complex conjugate of the reference function for accurate focusing [24,25], but its efficiency will decrease dramatically with increased range cell migration (RCM) caused by long dwell time and therefore, it is not suitable for the GNSS-based bistatic SAR.

In this paper, we consider the most complex geometry topology with non-parallel transmitter (GNSS satellites) and receiver (airplane) trajectories as well as unequal platform velocities, and propose a general imaging algorithm with bulk range cell migration correction (RCMC) and modified hybrid correlation processing. In the proposed algorithm, the bulk RCMC is employed to remove the phase resulted from RCM and range-azimuth coupling at the reference range; after this operation, all targets except for the reference one are coarsely focused; then, a modified hybrid correlation operation is performed with a short processing window in the range direction to compensate for the residual phase errors.

In order to demonstrate the validity and feasibility of our proposed imaging formation algorithm, points targets simulations are carried out in this paper. The receiver is assumed to fly in a straight line, without any atmospheric turbulence during its flight. For motion error compensation, which is outside the scope of this paper, adequate processing algorithms about bistatic SAR motion compensation have been proposed [16,26,27] and they are relatively mature nowadays. Besides, other receiver and atmospheric errors (like clock slippage and local oscillator) compensation methods have been proposed in [16]. In the simulations, firstly, the comparison of simulation results of our proposed and traditional BPA are presented to prove the effectiveness of our methods. Furthermore, the results of computational time demonstrate that our proposed algorithm is more efficient. Then, the imaging results and qualities of point targets in different locations are carried out to prove the robustness of the proposed algorithm on full-scene images. 

The organization of this paper is as follows: Section 2 provides the geometric configuration and echo signal model of GNSS-based bistatic SAR, followed by detailed analysis of the 2-D point target spectrum and system resolution in Section 3. The general imaging algorithm is proposed in Section 4, supported by simulation results in Section 5. Conclusions are drawn in Section 6.

## 2. Signal Model of GNSS-Based Bistatic SAR

**Figure 1:**
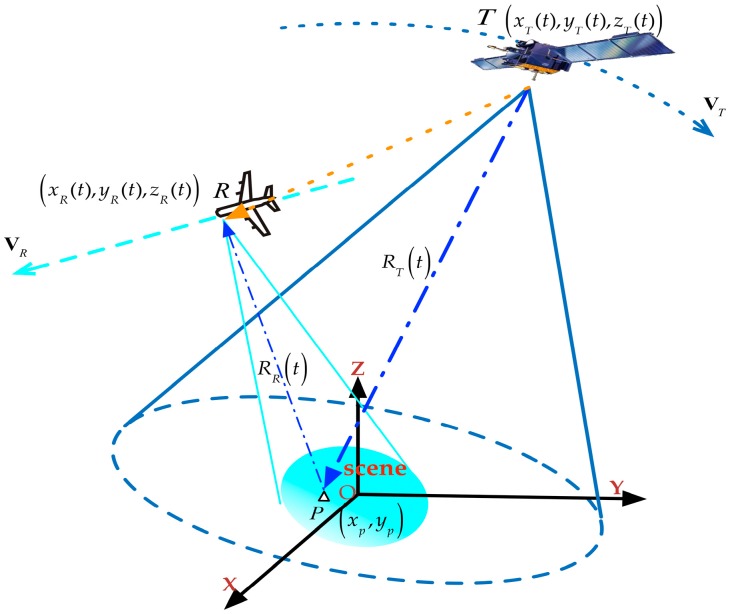
Geometric configuration of Global Navigation Satellite System (GNSS)-based bistatic Synthetic Aperture Radar (SAR).

Figure 1 shows the geometric configuration of GNSS-based bistatic SAR, where the origin *O* is set to be the scene center, the X-axis points to the East direction and the Y-axis points to the North. *P*(*x_p_*,*y_p_*) is an arbitrary point target in the imaging area (the targets are assumed to be located on the XOY plane). T and R are the transmitter (GNSS) and receiver (airplane), respectively. The transmitter is located at (*x_T_*(*t*)*,y_T_*(*t*)*,z_T_*(*t*)) with the velocity vector **V**_T_(*V_Tx_*,*V_Ty_*,*V_Tz_*), while the receiver at (*x_R_*(*t*),*y_R_*(*t*),*z_R_*(*t*)) has a velocity vector given by **V**_R_(*V_Rx_*,*V_Ry_*,*V_Rz_*). *R_R_*(*t*) and *R_T_*(*t*) denote receiver and transmitter slant range history, respectively.

The instantaneous slant range *R_T_*(*t*) and *R_R_*(*t*) for target *P* can be expressed as

(1) $$\begin{array}{l} R_{T}\left(t\right)=\sqrt{{\left[x_{T}\left(t\right)-x_{p}\right]}^{2}+{\left[y_{T}\left(t\right)-y_{p}\right]}^{2}+z_{T}^{2}\left(t\right)}\\ R_{R}\left(t\right)=\sqrt{{\left[x_{R}\left(t\right)-x_{p}\right]}^{2}+{\left[y_{R}\left(t\right)-y_{p}\right]}^{2}+z_{R}^{2}\left(t\right)} \end{array}$$

The range history *R*(*t*) is the sum of *R_T_*(*t*) and *R_R_*(*t*)

(2) $$R\left(t\right)=R_{T}\left(t\right)+R_{R}\left(t\right)$$

**Figure 2:**
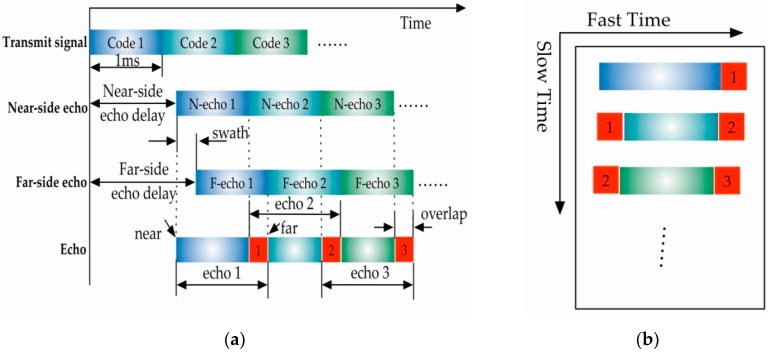
Structures of the GNSS-based bistatic SAR echo signals: (**a**) echo signal structure; and (**b**) 2-D layout of the echoes.

As the traditional SAR system transmits pulse signals, the train of echo signals can be divided into a 2-D vector in memory by the opening and closing of the receiving-window. However, for GNSS-based bistatic SAR, the transmitted signal frequency is fixed, and the receiving-window is open all the time. Each echo of the transmitted GNSS code begins from the near-side target echo and ends by the far side target echo, and there is an overlap between adjacent GNSS code echoes as indicated by the red parts shown in Figure 2a, which are shared by the far-side echo of the previous GNSS code and the near-side echo of the next GNSS code. Assuming that the receiver clocks are synchronized with the GNSS satellites by a tracking algorithm [28], the 2-D echo can be generated as shown in Figure 2b. If we just remove the overlapped part of the echo signal, the effective illuminated time of target from different range would be uneven, which would limit the application of GNSS-based bistatic SAR system. Generally speaking, as the length of aliasing part is significantly short enough and after imaging process with the unmatched parameters, the power of ghost target caused by the aliasing part is relatively small so that can be ignored. However, the valid range part still needs to be removed after the imaging process.

As a series of continuous wave signals are transmitted by GNSS-based bistatic SAR, its echo actually forms a one-dimensional vector. Assuming that encoding is used in each transmitted pulse *g*(*t*), *g*(*t*) can be expressed as

(3) $$g\left(t\right)=a\left(t\right)\cos\left(2\pi f_{0}t+\theta_{0}\right)$$

where *a*(*t*), *f*_0_ and *θ*_0_ denote the primary GNSS ranging code envelope, signal carrier frequency and initial signal phase, respectively. Then, the transmitted signal *p*(*t*) can be expressed as

(4) $$p\left(t\right)=\sum_{n=-\infty }^{\infty }g\left(t-nT\right)$$

where *T* is the code period. Hence, the echo *s*(*t*) reflected from the target *P* can be modeled as

(5) $$s\left(t\right)=\sigma p\left[t-\frac{R\left(t\right)}{c}\right]=\sum_{n=-\infty }^{\infty }\sigma g\left[t-nT-\frac{R\left(t\right)}{c}\right]$$

where *σ* is target radar cross section (RCS), and *c* is the speed of light. Here, we assume that the transmitter antenna pattern gain is constant to all illuminated targets and the receiver antenna pattern is a rectangular window function. Then, the echo *s*(*t*) after quadrature demodulation can be written as

(6) $$s\left(t\right)=\sum_{n=-\infty }^{\infty }\sigma a\left[t-nT-\frac{R\left(t\right)}{c}\right]\exp\left\{-j2\pi \frac{R\left(t\right)}{c}\right\}$$

where the constant phase term has been ignored. Let *R*(*nT*) ≈ *R*(*t*), and set *η* = *nT* + *τ* [24]. *s*(*t*) can be written in a 2-D form

(7) $$s\left(\eta ,\tau \right)=\sigma a\left[\tau -\frac{R\left(t\right)}{c}\right]\exp\left\{-j2\pi \frac{R\left(\eta \right)}{c}\right\}$$

where *η* and *τ* denote the slow-time and fast-time, respectively, and τ ∈ [*−T/2,T/2*]. 

However, as shown in Figure 2b, the valid range part should be removed after the imaging process. Thus, in the final non-aliased image, *τ* falls within the following range

(8) $$\tau \in \left[-\frac{R_{swath}}{2c},\frac{R_{swath}}{2c}\right]$$

where *R_swath_* is the range swath.

## 3. GNSS-Based Bistatic SAR Signal Analysis

In this section, formulation of the 2-D spectrum for a point target is first derived in detail and then system resolution of the GNSS-based bistatic SAR is obtained, followed by a suggested criterion for choosing opportunistic signal sources.

### 3.1. 2-D Point Target Spectrum

Applying FFT with respect to *τ*, the echo signal *s*(*η*, *τ*) is transformed into the range frequency domain, yielding

(9) $$\begin{array}{ll} S_{r}\left(\eta ,f_{\tau }\right) & =\int s\left(\eta ,\tau \right)\exp\left\{-j2\pi f_{\tau }\tau \right\}d\tau \\ & =\sigma A\left(f_{\tau }\right)\exp\left\{-j2\pi \frac{\left(f_{0}+f_{\tau }\right)R\left(\eta \right)}{c}\right\} \end{array}$$

where A(·) denotes the spectral function of the range code signal, and *f_τ_* and *f_0_* represent the range frequency and signal carrier frequency, respectively. Then, to obtain 2-D spectrum of the signal, the azimuth FFT is applied as

(10) $$\begin{array}{ll} S_{2D}\left(f_{\eta },f_{\tau }\right) & =\int S_{r}\left(\eta ,f_{\tau }\right)\exp\left\{-j2\pi f_{\eta }\eta \right\}d\eta \\ & =\sigma A\left(f_{\tau }\right)\int S_{r}\left(\eta ,f_{\tau }\right)\exp\left\{-j2\pi \frac{\left(f_{0}+f_{\tau }\right)R\left(\eta \right)}{c}-j2\pi f_{\eta }\eta \right\}d\eta \end{array}$$

where *f_η_* is the azimuth frequency. By applying the principle of stationary phase (POSP) [29], we have

(11) $$2\pi \frac{\left(f_{0}+f_{\tau }\right)}{c}\cdot \frac{dR\left(\eta \right)}{d\eta }+2\pi f_{\eta }=0$$

It is difficult to obtain an explicit solution for Equation (11). However, the GNSS-based bistatic SAR signal exhibits a relatively small bandwidth compared to the traditional SAR system, and its resolution is very low. Therefore, by using the series reversion method in [18,30], the 2-D point target spectrum is approximately given by

(12) $$\begin{array}{ll} S_{2D}\left(f_{\eta },f_{\tau }\right) & =\sigma A\left(f_{\tau }\right)\exp\left\{-j\frac{2\pi \left(f_{0}+f_{\tau }\right)r}{c}\right\}\\ & \cdot \exp\left\{j2\pi \cdot \frac{1}{2f_{r}}\cdot q\left(f_{\tau }\right)\cdot {\left[\frac{f_{d}}{q\left(f_{\tau }\right)}+f_{\eta }\right]}^{2}\right\}\\ & \cdot \exp\left\{j2\pi \cdot \frac{f_{3}}{6f_{r}^{3}}\cdot q^{2}\left(f_{\tau }\right)\cdot {\left[\frac{f_{d}}{q\left(f_{\tau }\right)}+f_{\eta }\right]}^{3}\right\}\\ & \cdot \exp\left\{j2\pi \cdot \frac{3f_{3}^{2}-f_{r}f_{4}}{24f_{r}^{5}}\cdot q^{3}\left(f_{\tau }\right)\cdot {\left[\frac{f_{d}}{q\left(f_{\tau }\right)}+f_{\eta }\right]}^{4}\right\} \end{array}$$

where $q\left(f_{\tau }\right)=\frac{f_{0}}{f_{0}+f_{\tau }}$, *r* is the target slant range at Doppler center time, and *f_d_*, *f_r_*, *f_3_* and *f_4_* denote the Doppler parameters, as given in the following Taylor series expansion

(13) $$R\left(\eta \right)=r+\lambda \left(f_{d}\eta +\frac{1}{2!}f_{r}\eta^{2}+\frac{1}{3!}f_{3}\eta^{3}+\frac{1}{4!}f_{4}\eta^{4}\right)$$

All the coefficients can be evaluated at the aperture center, *i.e.*,

(14) $$\begin{array}{l} f_{d}=\frac{1}{\lambda }{\left[\frac{dR_{T}\left(\eta \right)}{d\eta }+\frac{dR_{R}\left(\eta \right)}{d\eta }\right]|}_{\eta =0}\;,\;f_{r}=\frac{1}{\lambda }{\left[\frac{dR_{T}^{2}\left(\eta \right)}{d\eta^{2}}+\frac{dR_{R}^{2}\left(\eta \right)}{d\eta^{2}}\right]|}_{\eta =0}\\ f_{3}=\frac{1}{\lambda }{\left[\frac{dR_{T}^{3}\left(\eta \right)}{d\eta^{3}}+\frac{dR_{R}^{3}\left(\eta \right)}{d\eta^{3}}\right]|}_{\eta =0}\;,\;f_{4}=\frac{1}{\lambda }{\left[\frac{dR_{T}^{4}\left(\eta \right)}{d\eta^{4}}+\frac{dR_{R}^{4}\left(\eta \right)}{d\eta^{4}}\right]|}_{\eta =0} \end{array}$$

Furthermore, the maximum values of the cubic and quartic phase terms in Equation (12) are

(15) $$\begin{array}{c} \phi_{3}\approx \left|\frac{\pi f_{3}B_{a}^{3}}{24f_{r}^{3}}\right|\approx \left|\frac{\pi f_{3}T_{s}^{3}}{24}\right|\\ \phi_{4}\approx \left|\frac{\pi \left(3f_{3}^{2}-f_{r}f_{4}\right)B_{a}^{4}}{192f_{r}^{5}}\right|\approx \left|\frac{\pi \left(3f_{3}^{2}-f_{r}f_{4}\right)T_{s}^{4}}{192f_{r}}\right| \end{array}$$

where *B_a_ = f_r_T_s_* is the Doppler bandwidth and *T_s_* is the synthetic aperture time.

**Figure 3:**
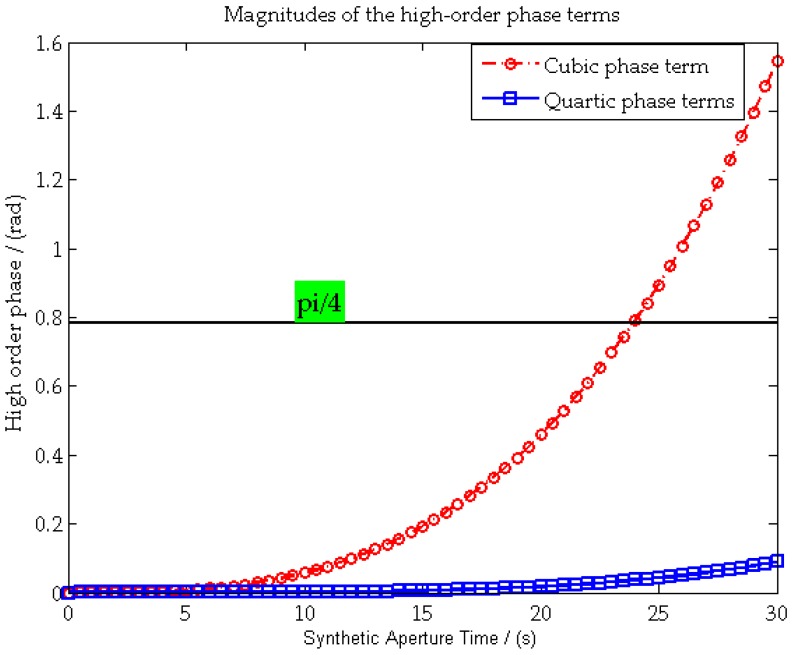
Maximum phase values of the cubic and quartic phase terms in the 2-D spectrum.

Figure 3 shows the changing curve of *φ*_3_, *φ*_4_ with respect to *T_s_*, with the parameters listed in Section 5. As shown in Figure 3, both *φ*_3_ and *φ*_4_ are smaller than π/4 (black dash lines) when *T_s_* is smaller than 24 s. For a general airplane receiver platform, such an upper bound for *T_s_* is easily satisfied, and the fourth order range history is sufficient for accurate focusing. For some other long dwell time applications, like a fixed receiver [8], a higher order range history is necessary.

### 3.2. System Resolution of GNSS-Based Bistatic SAR

Azimuth resolution and range resolution are important parameters of a GNSS-based bistatic SAR system. The general analysis approaches for spatial resolution of bistatic SAR have been presented in [31,32], and the generalized ambiguity function and point-spread function of GNSS-based multistatic SAR have been given in [12]. Based on this literature, the system resolution of GNSS-based bistatic SAR with a general configuration is analyzed in this section.

According to the principle of gradient method [31,33], the directions of range and azimuth resolutions are

(16) $$u_{a}=\frac{grad\left[f_{d}^{\left(x,y\right)}\left(0\right)\right]}{\left|grad\left[f_{d}^{\left(x,y\right)}\left(0\right)\right]\right|},\;\;u_{r}=\frac{grad\left[R^{\left(x,y\right)}\left(0\right)\right]}{\left|grad\left[R^{\left(x,y\right)}\left(0\right)\right]\right|}$$

where *f_d_* is the Doppler frequency and it can be found by differentiation of the range history with respect to *η*. *grad*(·) is the gradient function. The superscript (*x*,*y*) represents the target located at (*x*,*y*). Unlike the traditional monostatic SAR system, the range and azimuth resolution vectors of GNSS-based bistatic SAR are not orthogonal, and even almost in parallel with each other in some cases. The angle *θ* between the two vectors can be calculated by

(17) $$\theta =a\cos\left(u_{a}\cdot u_{r}\right)$$

**Figure 4:**
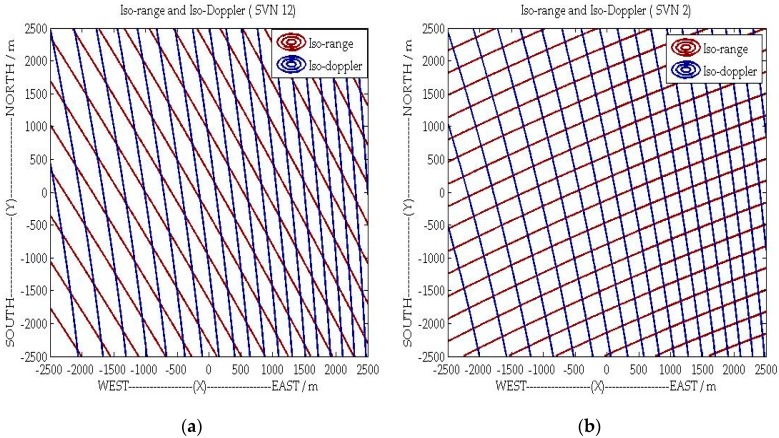
The Iso-range and Iso-Doppler lines of: (**a**) SVN 12; and (**b**) SVN 2.

**Figure 5:**
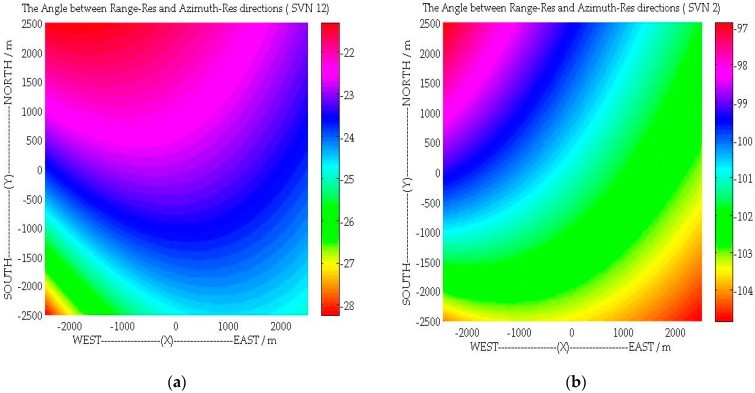
The Angle between the directions of Range and Azimuth resolutions: (**a**) SVN12; and (**b**) SVN 2.

Figure 4 shows the iso-range, iso-Doppler lines and the angle *θ* for a given area, with the parameters listed in Section 5. For the GPS SVN 12 satellite, as shown in Figure 4a and Figure 5a, the gradients of iso-range and iso-Doppler lines are nearly in parallel, which will have a negative impact on 2-D target detection after imaging. However, as demonstrated by Figure 4b and Figure 5b, the direction of range resolution is approximately perpendicular to the direction of azimuth resolution with the geometry topology of SVN 2.

**Figure 6:**
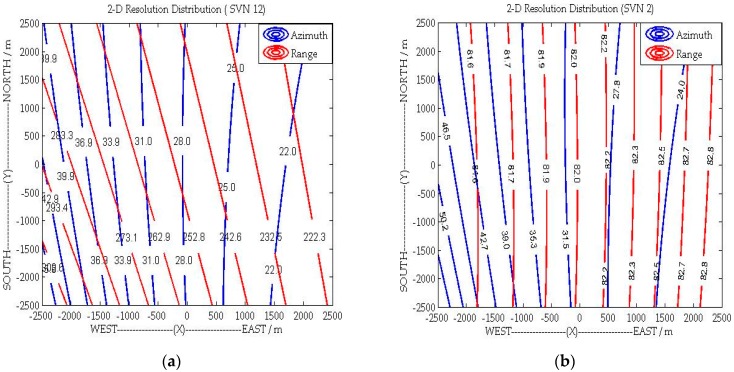
The 2-D resolution distribution of GNSS-based bistatic SAR: (**a**) SVN12; and (**b**) SVN 2.

Therefore, to obtain better 2-D resolutions, the best transmitter signal source should be chosen firstly among all visible GNSS satellites. Furthermore, according to the gradient of range history and Doppler frequency, the range and azimuth resolution can be obtained by [31]

(18) $$\rho_{a}=\frac{1}{\left|grad\left[f_{d}^{\left(x,y\right)}\left(0\right)\right]\right|}\cdot \frac{1}{T_{s}},\rho_{r}=\frac{1}{\left|grad\left[R^{\left(x,y\right)}\left(0\right)\right]\right|}\cdot \frac{c}{B_{w}}$$

where *B_w_* is the signal bandwidth. To understand the spatial variant 2-D resolution clearly, Figure 6a,b shows the 2-D resolution distribution with the configurations of GPS SVN12 and GSP SVN 2. As shown in Figure 6, unlike the monostatic SAR, the azimuth and range resolutions are spatially variant and both of them depend on the specific system bistatic geometry [31].

## 4. A General Imaging Algorithm

Based on the analysis in Section 3, a novel general imaging algorithm is proposed in this section. There are two major challenges for GNSS-based bistatic SAR imaging. The first is the intricate range history caused by the specific system geometry. In general, the transmitter and the receiver’s movements are independent, and their trajectories and speeds are essentially different. As a result, the 2-D spectrum of GNSS-based bistatic SAR is extremely complicated, as shown in Equation (12), and the solutions to range cell migration correction are therefore more complex. The second one is the long dwell time. In GNSS-based bistatic SAR system, long dwell time is necessary to achieve a reasonable azimuth resolution and sufficient image SNR for radar applications, which leads to large range migration and extremely big data size.

**Figure 7:**
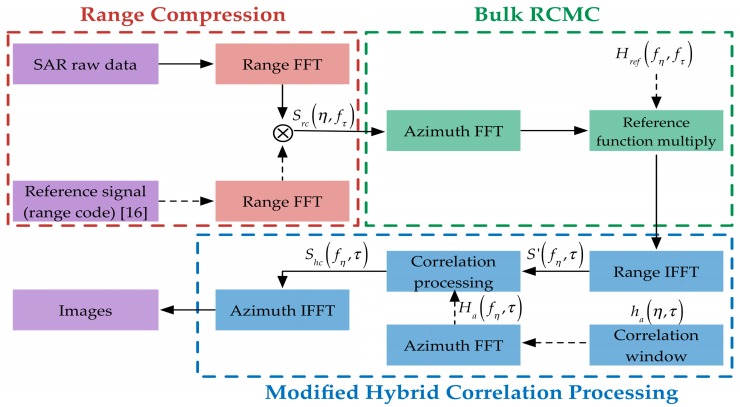
Block diagram of the proposed imaging algorithm.

Based on the aforementioned discussion, a novel general imaging algorithm based on hybrid correlation processing is derived. The block diagram of the proposed algorithm is shown in Figure 7. There are three parts: the first part is range compression, which is carried out to implement matched filtering in the range direction; the second part is bulk compression, which is used to decrease range migration and focus the targets at reference range; the last part is performing a modified hybrid correlation processing to acquire an accurate focused image.

### 4.1. Range Compression

To maximize signal-to-noise ratio and obtain fine range resolution of the sensed objects, range compression processing is performed. As in a single GNSS constellation system there are over 20 GNSS satellites and at least six to eight illuminating the same scene, the receiver of GNSS-based bistatic SAR actually records all the visible satellite signals. Therefore, the echo of GNSS-based bistatic SAR is the superposition of different reflected signal echoes. The GNSS network uses a high-rate pseudo random noise (PRN) sequence for each satellite. As a result, the PRN codes have particularly excellent auto-correlation and cross-correlation properties, and these cross-correlation values are so small that they usually can be ignored [34]. Therefore, the corresponding PRN code could be employed as the matched filter. Furthermore, after range compression, the reflected signals from different visible GNSS satellites are separated.

Range compression can be realized in the azimuth time range frequency domain via a range FFT on raw data and reference signal, matched filter multiplication and a range IFFT. The reference signal is determined by pseudo PRN sequence of the chosen satellite, and it can be conducted by the signal synchronization algorithm [16]. After matched filtering, the signal can be modeled as follows:

(19) $$S_{rc}\left(\eta ,f_{\tau }\right)=\sigma F_{f}\left(f_{\tau }\right)\exp\left\{-j2\pi \frac{\left(f_{0}+f_{\tau }\right)R\left(\eta \right)}{c}\right\}$$

where *F_f_*(·) represents the spectrum of the auto-correlation function *F_τ_*(·) between the echo data and reference signal. It is also highlighted that, if the aggregate E5 signal of Galileo system is used, range signal suppression technology should be adopted after range compression [35].

### 4.2. Bulk RCMC

For a GNSS-based bistatic SAR system, the imaging operation has to deal with a huge amount of raw data due to the large RCM. As a result, it will lead to a very high computational load and low efficiency for traditional imaging algorithms. The total RCM can be divided into two parts: bulk RCM and residual RCM. Bulk RCM represents the RCM of a reference target, while the remaining component of RCM is the residual RCM. Therefore, bulk RCMC is performed in the proposed algorithm to decrease the range migration and enhance the processing efficiency.

It starts with an azimuth FFT to transform the signal into the 2-D frequency domain. Then, multiplication with the reference function is performed to remove the bulk RCM, the frequency modulation in azimuth and range-azimuth coupling at the reference slant range. According to the 2-D spectrum in Equation (12), the reference function is given by

(20) $$\begin{array}{ll} H_{ref}\left(f_{\eta },f_{\tau }\right) & =\exp\left\{j\frac{2\pi \left(f_{0}+f_{\tau }\right)r}{c}\right\}\\ & \cdot \exp\left\{-j2\pi \cdot \frac{1}{2f_{r}}\cdot q\left(f_{\tau }\right)\cdot {\left[\frac{f_{d}}{q\left(f_{\tau }\right)}+f_{\eta }\right]}^{2}\right\}\\ & \cdot \exp\left\{-j2\pi \cdot \frac{f_{3}}{6f_{r}^{3}}\cdot q^{2}\left(f_{\tau }\right)\cdot {\left[\frac{f_{d}}{q\left(f_{\tau }\right)}+f_{\eta }\right]}^{3}\right\}\\ & \cdot \exp\left\{-j2\pi \cdot \frac{3f_{3}^{2}-f_{r}f_{4}}{24f_{r}^{5}}\cdot q^{3}\left(f_{\tau }\right)\cdot {\left[\frac{f_{d}}{q\left(f_{\tau }\right)}+f_{\eta }\right]}^{4}\right\} \end{array}$$

where *R_ref_* is reference range, and *f_d_re__f_*, *f_r_ref_*, *f_3_ref_*, and *f_4_ref_* are the corresponding Doppler parameters.

After the bulk RCM component is removed and the target phase at the reference range is compensated, a residual phase still exists for targets at other range bins and differential RCM is still present. The residual RCM is range dependent and is much smaller than the bulk RCM.

### 4.3. Modified Hybrid Correlation Processing

The bulk RCMC is introduced as a coarse focusing stage in Section 4.2, and the RCM is reduced accordingly. However, the form of target range history is changed after performing the bulk RCMC. Therefore, the traditional hybrid correlation processing is not suitable for differential focusing any more. In this stage, a modified hybrid correlation processing is proposed due to the different range history. To correct the residual RCM for all targets, a processing window in range direction is applied. The offset of the correlation window in time domain is

(21) $$h\left(\eta ,\tau \right)=F_{\tau }\left[\tau -\frac{R\left(\eta \right)-R_{ref}\left(\eta \right)}{c}\right]\exp\left\{-j2\pi \left[R\left(\eta \right)-R_{ref}\left(\eta \right)\right]\right\}$$

where *R_ref_*(*η*) is the range history of reference target. The signal after hybrid correlation processing can be expressed as

(22) $$S_{hc}\left(f_{\eta },\tau \right)=\sum_{i=0}^{m}S\prime \left[f_{\eta },\tau +\left(i-\frac{m}{2}+\left\lfloor \frac{R\left(f_{\eta }\right)-R_{ref}\left(f_{\eta }\right)}{c}f_{s}\right\rfloor \right)\right]\cdot H_{a}^{*}\left(f_{\eta },\tau \right)$$

where *S*’[·] represents the system signal after the range IFFT in the modified hybrid correlation processing stage, m is the length of correlation window in the range direction, *f_s_* is the range sampling rate, *H_a_*(*f_η_*, *τ*) is the azimuth FFT of correlation window, the superscript * represents the complex conjugate of *H_a_*(*f_η_*, *τ*), and ⌊⋅⌋ represents the floor function. As the bulk RCM has been removed at the bulk RCMC stage, a short correlation window in the range direction is used in the modified hybrid correlation processing, which means the efficiency of the proposed imaging algorithm is improved.

After hybrid correlation processing, the residual RCM and the residual phase error are completely corrected. Then, an azimuth IFFT is performed, leading to an accurately focused image.

## 5. Simulation and Discussions

**Figure 8:**
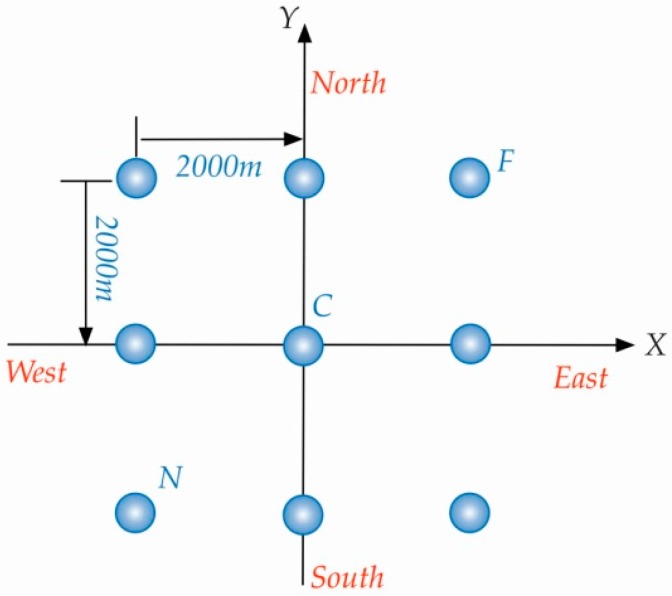
Distribution of the simulation scene.

In this section, the C/A code of GPS is adopted as the opportunistic signal for bistatic SAR simulation. As shown in Figure 4 and Figure 5, the geometric topology with satellite SVN 2 is favorable to achieve 2-D (range-azimuth) resolution at the same time. Therefore, the C/A code and motion state of the SVN 2 are used in the subsequent simulations to demonstrate the performance of the proposed general imaging algorithm. The simulation scene with point targets is illustrated in Figure 8 and the specific parameters are listed in Table 1, where we can see that the trajectories of transmitter and receiver are non-parallel, and their velocities are non-parallel and different.

### 5.1. Comparative Experiments and Analysis

**Figure 9:**
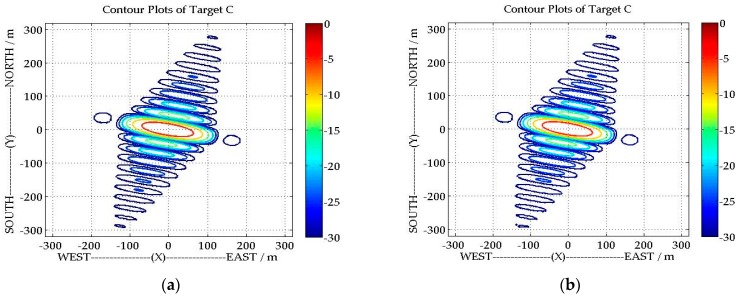
Contour plots of Target C: (**a**) imaging results of traditional Back Projection Algorithm (BPA); and (**b**) imaging results of our proposed algorithm.

To show the performance of the proposed algorithm, the imaging results of scene center C with the traditional BPA [16] and our proposed algorithm are shown in Figure 9. In order to compare their imaging results, the imaging results of the proposed algorithm have projected onto the ground plane (not the slant range plane), as the final imaging results of BPA are acquired in ground plane directly. Comparing these two imaging results, we would not be able to see a clear difference between them. Furthermore, the spatial resolution (azimuth resolution and range resolution), peak side lobe ratio (PSLR) and integrated side lobe ratio (ISLR) for target C are listed in Table 2. Both the imaging results and imaging qualities indicate that our proposed algorithm has been adequately and equally same effective with the traditional one. On the other hand, the computational time of traditional BPA is 172.9 s, with the parameter listed in Table 1. However, for our proposed algorithm, less CPU time is required (68.2 s), which means the proposed algorithm is more efficient. It should be noted that the simulations are carried out on a computer with Xeon E5649 2.53 GHz processors and 32 GB RAM, and all programs adopt the parallel codes (quad-core) in MATLAB.

### 5.2. Imaging Simulation Experiments and Analysis

**Figure 10:**
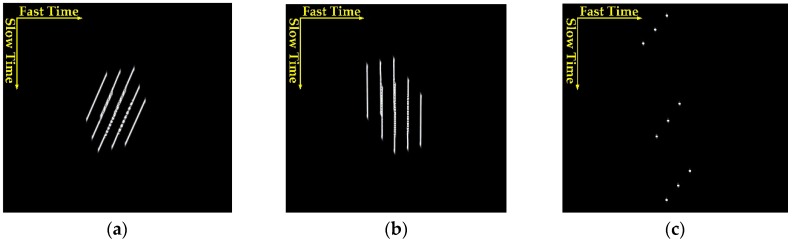
Processing results at different stages: (**a**) range compression result in range frequency azimuth time domain; (**b**) bulk RCMC result in range frequency azimuth time domain; and (**c**) final imaging result after azimuth compression.

To demonstrate the validity and feasibility of our proposed imaging formation algorithm, points targets imaging simulation experiments are carried out in this section. Figure 10 shows the processing results at different imaging stages. After the range compression stage, matched filtering in the range dimension is completed and the large RCM can been seen clearly in Figure 10a. In this case, the RCM is more than 100 range gates, which will reduce the efficiency of the imaging process, as a long correlation function (means *m* = 128 at least) has to be adopted in traditional hybrid correlation algorithms. Then, after performing the bulk RCMC, as shown in Figure 10b, the range migration is reduced significantly and only residual RCM exists. As a result, a correlation window with sixteen points (*m* = 16) in the range dimension is suggested for the general case, which indicates a notable saving in computational complexity by the proposed modified correlation operation. Even more savings can be achieved in long dwell time situations, such as a fixed receiver. The final imaging result is shown in Figure 10c, where the distortion caused by the specific geometric topology and mismatching 2-D resolution can be observed.

**Figure 11:**
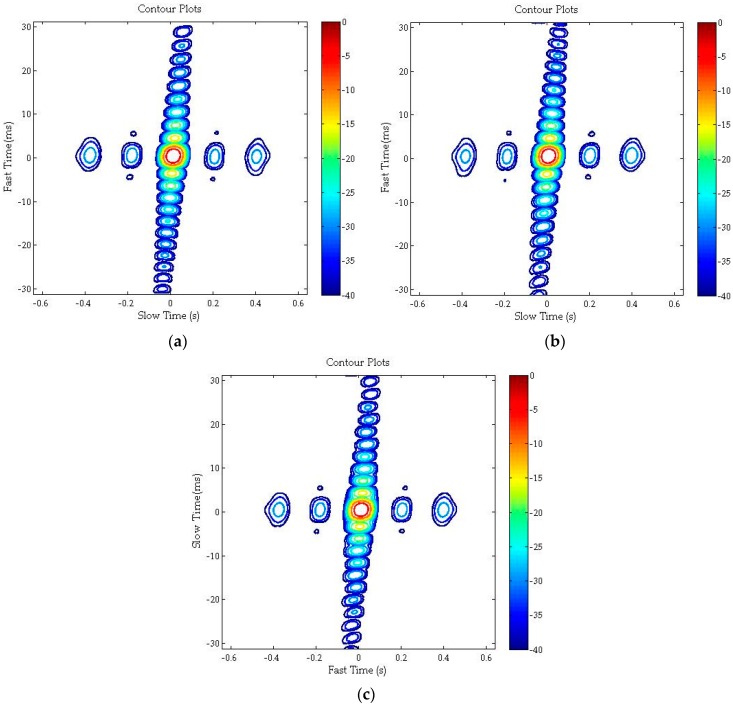
Interpolation imaging results of: (**a**) target N; (**b**) target F; and (**c**) target C.

**Figure 12:**
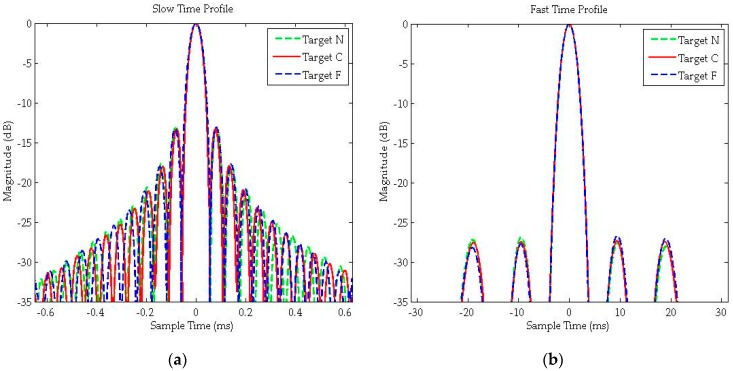
Interpolation imaging results of: (**a**) slow time profile; and (**b**) fast time profile.

Figure 11 shows the interpolated imaging results for targets N/C/F in slant range plane, and their 2-D profiles (fast time and slow time) are demonstrated in Figure 12. As shown in Figure 12a, the slow time profile is still a *sinc* envelope like traditional SAR results. However, as the non-designed transmit radar signal in GNSS-based bistatic SAR, the fast time profile is not a *sinc* function anymore and it is the envelope of the auto-correlation function of C/A code, as shown in Figure 12b. As demonstrated in both Figure 11 and Figure 12, low side-lobe in range dimension is achieved without any weighting operation. Furthermore, the spatial resolution (azimuth resolution and range resolution), widen ratio, PSLR and ISLR for targets N/C/F are listed in Table 3. In this specific geometric configuration, the azimuth resolution changes significantly in different spatial locations because of the rapidly changing range history. Finally, both the scene imaging results and imaging qualities of point targets have demonstrated excellent focusing performance of the proposed algorithm.

## 6. Conclusions

In this paper, a novel bistatic SAR system based on GNSS signals has been introduced, where the mechanism for its 2-D echo signal acquisition is analyzed and its 2-D point target spectrum model provided. To focus the echo data more accurately and efficiently, a general imaging algorithm without any interpolation processing was proposed. Due to the complex range history and large RCM in GNSS-based bistatic SAR, a bulk RCMC is applied first to remove the RCM of the reference target, avoiding a long and time-consuming range correlation window processing in the focusing stage; then, a modified hybrid correlation operation is carried out for accurate focusing of the echo data, with a much shorter correlation processing window (in most cases, sixteen points would be enough). As demonstrated by simulations results, the proposed GNSS-based bistatic SAR system works effectively and the imaging algorithm performs well. 

## Figures and Tables

**Table 1:** Main simulation parameters.

Parameters	Value
Receiver center position	(6, −25,5) km
Receiver velocity	(−30, 60, 0) m/s
Transmitter center position (SVN 2)	(1.0235, −1.5541, 1.2402) × 10^4^ km
Transmitter center position (SVN 12)	(−1.8028, 1.6145, 0.4677) × 10^4^ km
Transmitter center velocity (SVN 2)	(185.6, −2113.7, −1800.0) m/s
Transmitter center velocity (SVN 12)	(−2096.1, −730.7, −2321.8) m/s
Synthetic aperture time	10.0 s
Signal bandwidth (GPS C/A)	2.046 MHz
Signal wavelength	0.19 m
Sampling rate	5.0 MHz
*PRF*	100 Hz

**Table 2:** Imaging quality analysis of Comparative Experiments.

	Azimuth			Range		
	Resolution (m)	PSLR (dB)	ISLR (dB)	Resolution (m)	PSLR (dB)	ISLR (dB)
Traditional BPA	30.08	−13.30	−10.22	82.05	−27.34	−19.43
Proposed Algorithm	30.08	−13.30	−10.23	82.05	−27.34	−19.42

**Table 3:** Imaging quality analysis of point targets.

	Azimuth				Range			
	*ρ_a,m_* (m)	Widen Ratio	PSLR (dB)	ISLR (dB)	*ρ_r,m_* (m)	Widen Ratio	PSLR (dB)	ISLR (dB)
N	48.16	1.032	−13.26	−10.20	81.54	1.012	−27.09	−19.40
C	30.08	1.029	−13.30	−10.23	82.05	1.011	−27.34	−19.42
F	23.61	1.031	−13.24	−10.19	82.68	1.014	−26.99	−19.37

*ρ_a,m_ and *ρ_r,m_* denote the measured azimuth resolution and range resolution in *XOY* plane, and widen ratio represents the ratio between the measured and ideal resolution. The ideal resolution can be calculated by Equation (18) or the method in reference [31,32].
